# Advancing drug safety in psychiatry: insights from pharmacogenomics of hypersensitivity reactions

**DOI:** 10.3389/fphar.2025.1651898

**Published:** 2025-09-19

**Authors:** Hamid A. Alhaj, Jana Samara, Alyamama Alnamous, Rama Karima, Maha Saber-Ayad

**Affiliations:** ^1^ College of Medicine, University of Sharjah, Sharjah, United Arab Emirates; ^2^ Sharjah Institute for Medical Research, Sharjah, United Arab Emirates

**Keywords:** pharmacogenomics, drug hypersensitivity, psychiatric medications, HLA, CYP450, personalized medicine

## Abstract

Drug hypersensitivity reactions (DHRs) to psychiatric medications represent a significant clinical challenge, often resulting in treatment discontinuation, poor adherence, and compromised patient outcomes. Pharmacogenomics has emerged as a promising field for understanding and mitigating these adverse effects by identifying genetic predispositions that affect drug metabolism, immune responses, and individual susceptibility. This narrative review explores the multifaceted mechanisms underlying DHRs, with a focus on immunological pathways, particularly T cell-mediated responses, drug metabolite formation, and genetic risk factors. Among these, human leukocyte antigen (HLA) alleles and polymorphisms in cytochrome P450 (CYP450) enzymes are critical contributors to hypersensitivity development. We provide a comprehensive analysis of pharmacogenomic associations with commonly prescribed psychiatric drugs, including anticonvulsants (e.g., carbamazepine, lamotrigine), selective serotonin reuptake inhibitors (SSRIs), and novel agents such as vortioxetine, psilocybin, and esketamine. Additionally, we examine antipsychotics, including clozapine and newer agents like aripiprazole, brexpiprazole, and cariprazine, highlighting specific gene-drug interactions and known risk alleles such as HLA-B*15:02, HLA-A*31:01, and variants in *CYP2D6* and *CYP1A2*. These findings underscore the value of pharmacogenomic testing in predicting and preventing serious DHRs, such as Stevens-Johnson Syndrome, toxic epidermal necrolysis, agranulocytosis, and hepatotoxicity. The review also addresses clinical implementation, discussing the role of pre-emptive genetic screening, emerging guidelines from international consortia such as CPIC and DPWG, and real-world challenges, including variability in test accessibility, ethical concerns, and a lack of standardized protocols across regions. Recent advances in next-generation sequencing and multiomic approaches offer new opportunities to improve predictive accuracy and personalize psychiatric treatment further. Finally, we highlight the importance of population-specific research and global collaboration to close the evidence gap, particularly in underrepresented regions like the Middle East. This review emphasizes the transformative potential of pharmacogenomics in optimizing psychiatric drug therapy, enhancing safety, and ultimately improving patient-centered care.

## 1 Introduction

Drug hypersensitivity reactions (DHRs) represent a significant concern in the use of psychiatric medications, often leading to treatment discontinuation, prolonged hospitalization, and, in severe cases, life-threatening conditions such as Stevens-Johnson syndrome (SJS) or drug reaction with eosinophilia and systemic symptoms (DRESS) (Alchakee et al., 2022; Bousman et al., 2021; Wei et al., 2024). These adverse immune-mediated responses can occur with a wide range of psychotropic drugs, including anticonvulsants, antidepressants, and antipsychotics (Böhm and Cascorbi, 2016). The unpredictable nature of DHRs poses a major clinical challenge, as they are not dose-dependent and can manifest even after prior uneventful exposure (Elzagallaai and Rieder, 2022). Given the increasing reliance on pharmacotherapy in mental healthcare, understanding the mechanisms and risk factors of DHRs is crucial to improving patient safety and treatment outcomes.

Pharmacogenomics has emerged as a powerful tool in elucidating the genetic underpinnings of drug hypersensitivity, offering insights into interindividual variability in drug metabolism and immune response. Key genetic markers, such as human leukocyte antigen (HLA) alleles and polymorphisms in drug-metabolizing enzymes (e.g., *CYP2C19, CYP2D6*), have been strongly associated with DHR risk (Alchakee et al., 2022). For instance, the HLA-B*15:02 and HLA-A*31:01 variants are well-established predictors of carbamazepine-induced severe cutaneous reactions (Kaniwa and Saito, 2013). By integrating pharmacogenomic testing into clinical practice, clinicians may pre-emptively identify high-risk patients, enabling personalized drug selection and dose optimization to minimize adverse events.

This narrative review aims to synthesize current evidence on the pharmacogenomics of DHRs in psychiatric medications, focusing on genetic risk factors, mechanistic pathways, and clinical implications. We will examine HLA and non-HLA genetic associations across major psychotropic drug classes, evaluate the role of pharmacogenomic testing in preventing hypersensitivity reactions, and discuss challenges in implementation. Finally, we highlight future directions, including the integration of next-generation sequencing and global collaborative efforts to advance precision psychiatry. By bridging the gap between research and clinical practice, this review underscores the potential of pharmacogenomics to enhance drug safety and therapeutic efficacy in mental healthcare.

## 2 Mechanisms of drug hypersensitivity reactions

### 2.1 Immunological pathways involved in DHRs

Hypersensitivity reactions to psychiatric medications encompass a range of immune-mediated responses, often leading to undesirable side effects. These reactions are classified according to the Gell and Coombs system into four major types (Szegedi et al., 2023). Type I (Immediate, IgE-mediated), which is triggered by allergen-specific IgE antibodies bound to mast cells and basophils, leading to their degranulation and the release of mediators such as histamine, leukotrienes, prostaglandins, and Th2 cytokines (e.g., IL-4, IL-5, IL-13). This results in vasodilation, bronchospasm, and eosinophil recruitment (Kalesnikoff and Galli, 2008). Type II (Antibody-dependent cytotoxicity) involves IgG or IgM antibodies that recognize host cells altered by the drug, initiating complement activation and antibody-dependent cellular cytotoxicity (ADCC), ultimately causing cell lysis and tissue damage (Bajwa and Mohammed, 2025). Type III (Immune complex-mediated) occurs due to the deposition of circulating immune complexes, which activate complement and recruit neutrophils. This leads to the release of pro-inflammatory cytokines such as TNF-α and IL-1β, driving local inflammation and tissue injury (Uzzaman and Cho, 2012).

Type IV (Delayed-type hypersensitivity, T cell–mediated) is considered the leading mechanism in psychiatric drug hypersensitivity. This involves antigen-specific CD4^+^ T helper cells and CD8^+^ cytotoxic T cells activated by drug-modified peptides presented on MHC class II or I molecules, respectively. These T cells release a pro-inflammatory cytokine milieu—IFN-γ, TNF-α, IL-2, and IL-17—promoting immune cell recruitment and clonal expansion (Posadas and Pichler, 2007). Clinical manifestations include morbilliform rash, drug reaction with eosinophilia and systemic symptoms (DRESS), and severe cutaneous adverse reactions (SCARs) such as Stevens–Johnson syndrome (Hung et al., 2024).

In sensitized individuals, re-exposure to the drug may trigger an anamnestic Type I hypersensitivity reaction. Preformed drug-specific IgE or IgG can rapidly activate mast cells, causing urticaria, angioedema, or even anaphylaxis. Table 1 provides a summary of the major cutaneous adverse drug reactions associated with hypersensitivity to psychiatric drugs, along with their clinical characteristics.

**TABLE 1:** An outline of the principal cutaneous adverse drug reactions resulting from hypersensitivity to psychiatric medications.

Condition	Definition	Drug to scar interval	General symptoms	Skin features	BSA involved	Systemic involvement	Severity	Mortality rate	References
SJS	A rare, life-threatening, immune-mediated skin reaction characterized erythematous skin eruptions and extensive epidermal and mucosal detachment	7–21 days	Fever ≥38 °C, influenza-like syndrome, respiratory tract symptoms	Morbilliform or urticoid (raised, pruritic plaques “hives”) that rapidly progress to dusky, gray to red-violaceous atypical targetoid lesions, then flaccid blisters with full-thickness skin sloughing involving both skin and mucous membranes. Lesions are painful	<10%, toxic epidermal necrolysis	lips, mouth, pharynx, esophagus and gastrointestinal tract, eyes, genitals, and upper respiratory tract Liver, kidneys, lungs, bone marrow, and joints may be affected if patient is severely ill	often Severe (SCAR)	1%–5%	(Roujeau, 2005) Del Pozzo-Magaña and Liy-Wong (2024) Graudins et al. (2018) Mockenhaupt (2017) Harr and French (2010)
TEN	An immune-mediated, skin reaction that results in extensive blistering of the skin and mucous membranes (a more severe form of Stevens-Johnson syndrome)	7–21 days	Fever ≥38 °C, influenza-like syndrome, respiratory symptoms	morbilliform or urticoid (raised, pruritic plaques “hives”) that rapidly progress to dusky, gray to red-violaceous atypical targetoid lesions, then flaccid blisters with full-thickness skin sloughing involving both skin and mucous membranes. membranes. membranes. Lesions are painful	≥30%	lips, mouth, pharynx, esophagus and gastrointestinal tract, eyes, genitals, and upper respiratory tract Liver, kidneys, lungs, bone marrow, and joints may be affected if patient is severely ill	often Severe (SCAR)	25%–35%	Del Pozzo-Magaña and Liy-Wong (2024) Roujeau (2005) Mockenhaupt (2017) Harr and French (2010)
DRESS syndrome	Rare, potentially life-threatening adverse drug reaction caused by delayed hypersensitivity to medication	2–6 weeks	Fever ≥38 °C	Pink to red-brown macules and papules often start at the start in the axilla and groin, spreading and coalescing symmetrically across the body. Often spares the face and mucous membranes. Can be pruritic	usually involves >50%	facial edema, follicular accentuation (inflammation of the hair follicles), and systemic features (e.g., fever, lymphadenopathy, arthritis/arthralgias, multi-organ involvement, peripheral eosinophilia)	Severe (SCAR)	10%	Del Pozzo-Magaña and Liy-Wong (2024) Mockenhaupt (2017) Chowdhury et al. (2025)
AGEP	Adverse cutaneous reaction characterized by sterile pinpoint Non follicular pustules atop an erythematous background	24 h to 4 days	Fever ≥38 °C	Tiny Non follicular sterile pustules with underlying erythema that develop and spread rapidly and are accompanied by pruritus or burning sensation	variable 10% in mild to more than 30% in severe	Rare: desquamating skin, hepatomegaly, lymphadenopathy, liver injury, kidney injury, hypocalcemia, pleural effusions, respiratory distress, agranulocytosis, and even multiorgan involvement	Severe (SCAR)	<5%	(Moore et al., 2023) Del Pozzo-Magaña and Liy-Wong (2024) Mockenhaupt (2017)
Maculopapular rash	Morbilliform eruptions resemble measles, with widespread pink to red-brown macules and papules	7–14 days (timing can differ if previously sensitized)	Low-grade fever Malaise	Pink to red-brown macules and papules often start in the axilla and groin, spreading and coalescing symmetrically across the body. Often spares face and mucous membranes Can be pruritic	Usually involve <30% of body surface area	Rare. But may present with lymphadenopathy	Mild but may also be the initial presentation of a SCAR	Low	(Chowdhury et al., 2025) Del Pozzo‐Magaña and Liy‐Wong (2024) Muzumdar et al. (2019)
Urticarial toxidermia	Commonly called hives, a skin reaction characterized by weals, which are caused by the release of histamine and other vasoactive substances from mast cells	1 or 2 h or several days if delayed	Angioedema (can involve face, lips) Risk of anaphylaxis	Lesions are typically intensely pruritic and can induce a burning sensation	Variable 10% in mild to more than 30% in severe	Rare. Unless anaphylaxis	Mild to severe	Low	(Verheyden et al., 2020)
Fixed pigmented erythema (FPE)	An acquired type of hyperpigmentation that results from injury, inflammation, or procedures	24 h to a few days	Rarely	characterized by a single or multiple round to oval-shaped, red to brown macules	Variable 10% in mild to more than 30% in severe	Rare	Mild	Low	Del Pozzo‐Magaña and Liy‐Wong (2024) Mockenhaupt (2017)

SCAR, severe cutaneous adverse reaction; SJS, Stevens-Johnson syndrome; TEN, toxic epidermal necrolysis; DRESS, drug reaction with eosinophilia and systemic symptoms; HHV, human herpesvirus; EBV, Epstein-Barr virus; CMV, cytomegalovirus; AGEP, acute generalized exanthematous pustulosis.

### 2.2 Molecular basis of hypersensitivity reactions

The immune mechanisms underlying hypersensitivity reactions to drugs, including psychiatric medications, are complex and involve several proposed models that explain how small molecules can trigger immune responses.

#### 2.2.1 Hapten/pro-hapten model

In this classical model, drugs or their reactive metabolites—often too small to elicit an immune response alone—act as haptens by covalently binding to endogenous proteins, forming drug-protein conjugates. These neo-antigens are processed by antigen-presenting cells (APCs), which present drug-modified peptides on major histocompatibility complex (MHC) class I or II molecules to naïve T cells. If recognized as foreign, these peptide-MHC complexes can activate T cells, leading to an adaptive immune response (Adair et al., 2021).

#### 2.2.2 Pharmacological interaction with immune receptor (p-i) model

The p-i model proposes that certain drugs can directly, non-covalently bind to immune receptors such as HLA molecules or T cell receptors (TCRs), without requiring antigen processing or covalent modification. This reversible interaction can trigger T cell activation in a peptide-independent manner, bypassing classical antigen presentation pathways (Pichler, 2019).

#### 2.2.3 Altered self-repertoire model

This model, exemplified by abacavir hypersensitivity in individuals with HLA-B*57:01, demonstrates how a drug can bind within the peptide-binding groove of an HLA molecule, altering the repertoire of self-peptides presented to T cells. The immune system, now exposed to novel peptide-HLA complexes, may mistakenly identify these as foreign, thereby initiating an immune response against self (Hammond et al., 2021; Ostrov et al., 2012).

#### 2.2.4 Heterologous immunity model

This hypothesis is based on the concept of cross-reactivity in memory T cells. T cells previously primed by unrelated pathogens may possess TCRs capable of recognizing structurally similar drug-induced peptide-HLA complexes. In individuals with the appropriate HLA risk allele and a relevant infectious history, these cross-reactive memory T cells can be reactivated by the drug, resulting in an adverse immune response (Almeida et al., 2019; Pichler, 2019; White et al., 2015).

#### 2.2.5 Danger model

According to the danger model, immune activation is not solely dependent on antigen recognition but also on the presence of endogenous “danger signals” released during cellular stress, injury, or infection. These signals, such as damage-associated molecular patterns (DAMPs), can enhance APC activation and promote an immune response to otherwise tolerogenic drug antigens (Land, 2023; Pirmohamed et al., 2002).

**FIGURE 1:**
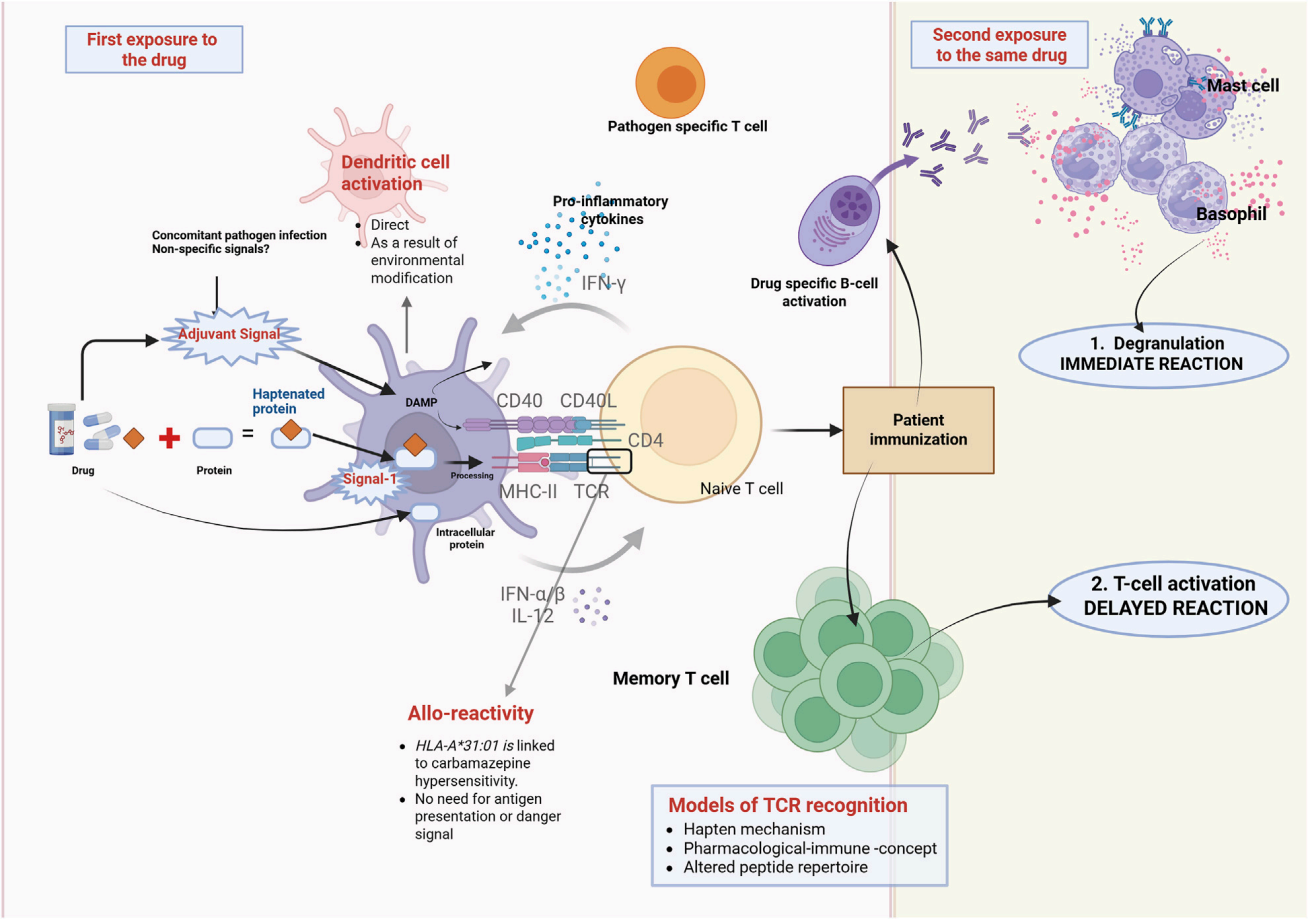
Overview of T cell activation and drug allergy from an immunological perspective. T cells are essential for drug allergies and fundamental to all immune-mediated hypersensitivity reactions. Chemically reactive drugs or drug metabolites can engage with the immune system and end up activating it *via* various mechanisms. The hapten hypothesis suggests that drugs attach covalently to proteins, creating a new antigen. Signal 1 refers to the identification of the modified antigen, presented by MHC molecules, to the TCR. To effectively stimulate T cell activation and patient immunization, co-stimulation (Signal 2) is necessary in addition to Signal 1. Therefore, in the absence of danger signals, Signal 2 does not get activated, resulting in immune tolerance. If the individual is re-exposed to the drug, they may experience a hypersensitivity reaction such as urticaria, angioedema, AGEP, DRESS, SJS, or TEN. In this context, mechanisms that contribute to memory T cell activation, alongside the hapten hypothesis, include the PI concept and altered peptide repertoire (modified from Azoury et al., 2018). Abbreviations: AGEP, DRESS, SJS, TEN. AGEP = Acute generalised exanthematous pustulosis, MHC = major histocompatibility, SJS = Stevens-Johnson syndrome, TCR = T-cell receptor, TEN = Toxic Epidermal Necrolysis.

Figure 1 illustrates the immunological overview of T cell activation in drug-induced hypersensitivity reactions.

## 3 Genetic factors influencing psychiatric drug hypersensitivity

The HLA system, located within the major histocompatibility complex (MHC) on the short arm of chromosome 6 (6p21.3), plays a central role in immune recognition and response. The HLA molecules enable the immune system to distinguish between ‘self’ and ‘non-self’ antigens—essential for complement activation, cytotoxic T cell function, and the coordination of both cellular and humoral immunity (Crux and Elahi, 2017). While vital for host defense, the HLA system is also implicated in various autoimmune and inflammatory diseases, as well as hypersensitivity reactions to drugs.

The HLA genes are categorized into three primary classes, namely: Class I (HLA-A, -B, -C): Present intracellular peptides to CD8^+^ cytotoxic T lymphocytes, Class II (HLA-DR, -DQ, -DP): Present extracellular antigens to CD4^+^ helper T cells, and Class III: Encode immune-related proteins such as components of the complement system (Crux and Elahi, 2017). Importantly, the HLA region is the most polymorphic in the human genome, with over 9,000 alleles identified to date (Anaya et al., 2013). These polymorphisms, particularly in the peptide-binding domains, significantly influence antigen presentation and subsequent T cell activation.

Certain drugs can bind directly to specific HLA molecules or to HLA-bound peptides, a mechanism described by the pharmacological interaction (p-i) hypothesis. This non-covalent interaction can activate T cells independently of antigen processing, leading to hypersensitivity reactions (Meng et al., 2018).

Ethnic diversity plays a critical role in the distribution of HLA alleles, directly influencing individual susceptibility to drug hypersensitivity. This variation underscores the importance of population-based pharmacogenomics in guiding safe drug prescribing (Alfirevic and Pirmohamed, 2010). For instance, alleles associated with severe cutaneous adverse reactions (SCARs), such as Stevens–Johnson syndrome (SJS) and toxic epidermal necrolysis (TEN), are expressed at differing frequencies across populations in the Middle East and North Africa (MENA), including the United Arab Emirates (UAE) (Masmoudi et al., 2022).

The Clinical Pharmacogenetics Implementation Consortium (CPIC) has identified several HLA alleles linked to psychiatric and antiepileptic drug hypersensitivity:

• HLA-B*15:02 – Strongly associated with carbamazepine-induced SJS/TEN. Though rare in Near Eastern populations (frequency: 0.0002), its clinical significance remains high due to the severity of reactions. *In addition, a strong association has also been reported between HLA-B15:02* and phenytoin-induced SJS/TEN (Locharernkul et al., 2008).
• HLA-B*57:01 – Associated with hypersensitivity to bupropion, with a higher regional frequency (0.0234) (Pavlos et al., 2012).
• HLA-A*31:01 – Also linked to carbamazepine hypersensitivity, with a regional frequency of 0.0111 (González-Galarza et al., 2015).

Whilst these data offer insight into regional allele prevalence, they lack the ethnicity-specific granularity necessary for precise pharmacogenetic recommendations. This highlights the urgent need for nation-specific studies in the MENA region, particularly in the UAE, to better understand HLA distribution and improve personalized drug safety. Currently, the UAE faces limitations in terms of resources and data availability related to HLA-associated drug hypersensitivity, underscoring the importance of integrating pharmacogenomic testing and research into clinical and public health practice. The ongoing Emirati Genome Program is expected to provide valuable resource for pharmacogenomics studies in the near future.

Table 2 summarizes the key associations between specific HLA gene variants and adverse hypersensitivity reactions to psychiatric and antiepileptic drugs. Understanding these gene–drug interactions is crucial for identifying patients at increased risk and guiding pharmacogenetic-informed prescribing to improve drug safety (Q. Wang et al., 2011).

**TABLE 2:** HLA Alleles Associated with Psychiatric Drug Hypersensitivity Reactions. The table summarizes some key associations between specific HLA gene variants and adverse hypersensitivity reactions to psychiatric and antiepileptic drugs.

HLA allele	Drugs	Clinical outcome(s)	Level of evidence	Guideline	References
HLA-B*15:02	Carbamazepine Phenytoin Lamotrigine	Stevens-Johnson Syndrome Toxic Epidermal Necrolysis Maculopapular Eruption	1A 1A 1A	Guideline	Wang et al. (2011) Locharernkul et al. (2008), Koomdee et al. (2017)
HLA-A*31:01	Carbamazepine	Maculopapular exanthema Hypersensitivity syndrome SJS/TEN	1A	Guideline	McCormack et al. (2011) Kim et al. (2011)
HLA-B*35:08 HLA-B*39:01 HLA-B*44:03 HLA-A*02:07 HLA-A*33:03	Lamotrigine	Maculopapular eruption (MPE)	3		Koomdee et al. (2017)
HLA-DRB1*07:01	Fluoxetine Sertraline	Severe Cutaneous Adverse Reaction “SCAR”	NA NA		Ahmed et al. (2021)
HLA-B*57:01	Bupropion	SJS TEN	NA		Pavlos et al. (2012)
HLA-DQB1*05:02	Clozapine	Agranulocytosis	3		Islam et al. (2022)
HLA-B*59:01	Clozapine	Myocarditis	NA		Islam et al. (2022)

## 4 Pharmacogenomics of specific psychiatric drugs

DHRs in psychiatric medications exhibit significant variability across drug classes, with distinct pharmacogenomic risk factors influencing susceptibility (Athanasiu et al., 2015; Cacabelos et al., 2013). Understanding these genetic associations is critical for risk stratification and personalized treatment approaches (Li et al., 2021). Pharmacogenomic research has identified key genetic markers for DHRs across major psychiatric drug classes, particularly for anticonvulsants and clozapine. However, gaps remain in understanding the genetic basis of hypersensitivity to newer antidepressants and some antipsychotics. Recent advances in psychopharmacology have introduced novel rapid-acting antidepressants, such as esketamine (a ketamine derivative) and psilocybin (a serotonergic psychedelic), offering new hope for treatment-resistant depression. However, their pharmacogenomic profiles and potential hypersensitivity risks remain understudied. Emerging evidence suggests that genetic variability in drug metabolism and receptor interactions may influence both therapeutic response and adverse reactions.

### 4.1 Anticonvulsants

Anticonvulsants, particularly carbamazepine and lamotrigine, are strongly associated with severe cutaneous adverse reactions (SCARs), including Stevens-Johnson syndrome (SJS) and toxic epidermal necrolysis (TEN). The HLA-B*15:02 allele is a well-established risk factor for carbamazepine-induced SCARs in East and Southeast Asian populations, leading to regulatory recommendations for pre-emptive genetic testing in these groups. Similarly, HLA-A*31:01 has been linked to carbamazepine hypersensitivity across diverse ethnicities, though with varying predictive strength. For lamotrigine, HLA-B*38:02 and HLA-B*07:02 have been implicated in European and Hispanic populations, though evidence remains less consistent than for carbamazepine. Additionally, polymorphisms in drug-metabolizing enzymes (e.g., EPHX1, UGT1A4) may influence lamotrigine metabolism and toxicity risk.

### 4.2 Antidepressants

Hypersensitivity reactions to antidepressants, while less common than with anticonvulsants, can still pose significant clinical challenges. Selective serotonin reuptake inhibitors (SSRIs) such as fluoxetine and sertraline have been associated with cutaneous reactions, with some evidence implicating HLA-DRB1*07:01 and *CYP2D6* poor metabolizer status in increased susceptibility (Ahmed et al., 2021). Emerging evidence suggests that pharmacogenomic testing may help identify individuals at increased risk of antidepressant-induced suicidality, a rare but serious adverse effect (Clayden et al., 2012).

The Hypothalamic-Pituitary-Adrenal (HPA) axis and specific neurotransmitter systems, particularly the serotonergic pathways, play a significant role in the pathophysiology of depression (Alhaj et al., 2008; Alhaj and McAllister-Williams, 2008; Andrews and Matthews, 2004; McAllister-Williams et al., 2014). Dysregulation of the HPA axis often results in hyperactivation and elevated cortisol levels, which can impair neuronal plasticity and contribute to mood disturbances (Calabrese et al., 2009). Similarly, alterations in neurotransmitter systems, especially dysfunction in serotonergic signaling, have been consistently associated with depressive symptoms. These abnormalities can affect emotional regulation, stress response, and cognitive processing, suggesting that both neuroendocrine and neurotransmitter mechanisms are interrelated contributors to the development and persistence of depression (Alhaj et al., 2012; McAllister-Williams et al., 2014).

Genetic variants in serotonergic pathways (e.g., *SLC6A4* serotonin transporter polymorphisms), HPA axis-related genes (e.g., *FKBP5*), and drug-metabolizing enzymes (e.g., *CYP2C19* and *CYP2D6*) have been associated with heightened vulnerability to suicidal ideation during antidepressant treatment (Clayden et al., 2012; De la Cruz-Cano, 2017). For instance, *FKBP5* gene polymorphisms affect the sensitivity and function of the glucocorticoid receptor (GR), because the protein encoded by the *FKBP5* gene functions as a co-chaperone of the GR receptor, thus serving as a negative regulator of the HPA axis. When paired with environmental factors like childhood trauma, the *FKBP5* gene polymorphisms are associated with dysregulation of the HPA axis, altered stress responses, and greater susceptibility to stress-related diseases like depression and PTSD. The negative feedback mechanism that ends the HPA stress response depends on *FKBP5*, and changes in the gene can make this feedback loop less effective (Malekpour et al., 2023). Moreover, poor metabolizers of *CYP2C19* may experience elevated drug levels, increasing agitation or emotional destabilization, while certain *SLC6A4* short-allele carriers may exhibit heightened emotional reactivity to SSRIs. Although current pharmacogenomic guidelines do not yet include suicidality risk prediction as a standard application, polygenic risk scores and integrated pharmacogenomic-pharmacodynamic models hold promise for future risk stratification.

Bupropion has been linked to hypersensitivity, possibly mediated by HLA-B*57:01 (Pavlos et al., 2012). Agomelatine’s metabolic pathway (primarily CYP1A2) may influence adverse drug reactions in slow metabolizers. Limited pharmacogenomic data exist on other antidepressants such as the serotonin modulator and stimulator vortioxetine. However, case reports suggest potential immune-mediated reactions, warranting further investigation into HLA and cytochrome P450 (e.g., *CYP2D6, CYP3A4*) influences.

Esketamine, an NMDA receptor antagonist used in treatment-resistant depression, undergoes primarily CYP3A4-mediated metabolism with secondary contributions from CYP2B6 (Langmia et al., 2022; Willemin et al., 2022), creating clinically relevant pharmacogenomic considerations as genetic polymorphisms in these enzymes may significantly alter drug clearance and exposure profiles. *CYP3A4* poor metabolizers (particularly those with CYP3A4*22 alleles) demonstrate reduced metabolic capacity that could prolong esketamine exposure, potentially exacerbating dose-dependent adverse effects including dissociation, hypertension, and hepatotoxicity, while functionally significant *CYP2B6* variants (notably the CYP2B6*6 haplotype associated with reduced enzyme activity) may further modulate therapeutic outcomes through altered ketamine metabolism pathways. Although current evidence has not established definitive HLA associations with esketamine hypersensitivity, pharmacovigilance reports of rare but clinically significant allergic-like reactions (including urticaria and angioedema) suggest potential immunogenetic components that warrant systematic investigation, particularly given esketamine’s structural similarity to known haptenic compounds and its increasing use in vulnerable psychiatric populations.

Psilocybin, a prodrug of psilocin, exerts its psychedelic effects primarily through 5-HT2A receptor agonism, with emerging evidence suggesting pharmacogenomic influences on both its therapeutic and adverse effects. While its metabolism involves deamination by monoamine oxidase (MAO) and glucuronidation *via* UGT1A10, the pharmacogenomic determinants of hypersensitivity reactions remain poorly characterized. Genetic variability in the 5-HT2A receptor gene (*HTR2A*), particularly the T102C polymorphism (rs6313), has been shown to modulate subjective psychedelic experiences, though its potential role in mediating adverse reactions requires further investigation. Metabolic pathways may present additional risk factors, as functional polymorphisms in *MAOA* (particularly the high-activity 3.5/4-repeat VNTR) and UGT1A10 could theoretically alter psilocin clearance rates, potentially influencing drug tolerance or hypersensitivity risk. Notably, while no HLA alleles have yet been conclusively linked to psilocybin reactions, the compound’s structural similarity to serotonin and reported cases of psychedelic-induced mast cell activation suggest possible immunogenetic mechanisms that warrant systematic study, particularly given the increasing therapeutic use of psilocybin in psychiatric disorders.

### 4.3 Antipsychotics

Antipsychotics exhibit a wide spectrum of hypersensitivity risks, with clozapine being the most extensively studied due to its well-documented associations with agranulocytosis and myocarditis (Vickers et al., 2022). The HLA-DQB1*05:02 allele has been strongly linked to clozapine-induced agranulocytosis, while HLA-B*59:01 shows association with myocarditis risk, particularly in Asian populations (Goldstein et al., 2014; Islam et al., 2022). However, global implementation of HLA screening remains inconsistent due to varying allele frequencies across ethnicities (Mahmood et al., 2021; Wilkinson et al., 2023). Furthermore, polymorphisms in *CYP1A2*, the primary metabolic pathway for clozapine, significantly influence plasma concentrations, with slow metabolizers at higher risk for both toxicity and efficacy failure.

Among second-generation antipsychotics, olanzapine hypersensitivity reactions (particularly cutaneous adverse events) have been anecdotally associated with HLA-B*38:02, though robust evidence remains limited (Pattanaik et al., 2021). Aripiprazole, metabolized predominantly by CYP2D6, demonstrates increased hypersensitivity risk in poor metabolizers who may accumulate supratherapeutic drug levels, potentially triggering immune-mediated reactions (Zhang et al., 2019). Emerging evidence suggests similar pharmacogenomic considerations for newer antipsychotics: brexpiprazole (also *CYP2D6*-dependent) shows altered pharmacokinetics in poor metabolizers (Elmokadem et al., 2022; Frederiksen et al., 2023). Cariprazine is mainly metabolized by CYP3A4 and hence may pose heightened hypersensitivity risks in patients with CYP3A4 inhibitors or genetic variants affecting metabolic capacity (Periclou et al., 2021; Szabó et al., 2024).

First-generation antipsychotics like haloperidol, while rarely causing hypersensitivity, may induce severe reactions in CYP2D6 poor metabolizers due to impaired drug clearance (Šimić et al., 2016). These findings underscore the critical need for pharmacogenomic-guided approaches in antipsychotic therapy to optimize both safety and efficacy, particularly for high-risk medications and vulnerable patient populations.

## 5 Determinants of drug hypersensitivity reactions beyond genetics

There is a myriad of general factors related to the drug and the patient, which interact to determine the occurrence and severity of the DHR. Drug-related factors include protein reactivity, the ability of the compound/drug to induce a danger/stress signal, the presence of a T-cell repertoire to recognize the antigen, and the frequency and route of the drug administration (Naisbitt et al., 2000).

Drugs of low molecular weight may directly react with proteins or undergo UV-light activation in the case of photosensitivity (bioactivation) to become protein-reactive. The hapten-protein bioconjugates formed through direct binding, bioactivation, or UV-light-dependent activation can be immunogenic; however, the mechanism of the immune response remains to be elucidated (Adair et al., 2021; Tailor et al., 2016). Interestingly, the hapten-protein complexes could be found in allergic and non-allergic individuals. On the other hand, some medications can induce or activate the stress/danger signals by activating the dendritic cells through increasing the expression of CD40. The damage-associated molecular pattern (DAMP) molecules, such as HMGB1, serve as a biomarker for adverse drug reactions (ADRs) (Carr and Pirmohamed, 2018). The HMGB1 serum levels are usually elevated in DRESS and SJS/TEN (Fujita et al., 2014; Nakajima, 2011), whereas IL-33 increases in early stages of TEN (Adachi et al., 2019). Whether the level of rise of HMGB1 can be used a biomarker for the severity of such reactions should be further evaluated.

Some models have been developed to recognize the drug-related hypersensitivity reactions using THP-1 cells and IL-8 production by mature dendritic cells (Kim and Naisbitt, 2016; Pallardy and Bechara, 2017).

Two important points are noteworthy; first, the presence of T-cell programming or a specific repertoire of T-cells (e.g., in the case of benzylpenicillin). In such case, the related peptides can be immunodominant and recognized by peripheral blood polymorphic Cells (PBMC) of the allergic patients, and can be used in immunization of such patients (Azoury et al., 2018). Second, in many cases, intermittent and repeated administrations of some drugs lead to more sensitization than uninterrupted treatment. Moreover, parenteral administration appears to be more sensitizing than the oral route.

In addition to genetics as a major determinant of DHR, several other patient-related factors exist. Drug-specific T-cell responses in allergic patients have been studied, in addition to the drug-responsive naïve T cells. To address specific T-cell responses to drugs using *ex vivo* samples from allergic patients, several protocols have been established, with the lymphocyte transformation test being the most frequently used assay (Naisbitt et al., 2014). Noteworthy, elderly patients and those on polypharmacy have a higher risk of developing allergic diseases (Cardona et al., 2011). The risk of DHR is also related to concurrent infections (Castrejon et al., 2010). Individual variations of T-regulatory cell number and the immune checkpoint expression (e.g., PD-1 and CTLA4) can explain the wide range of presentations of allergic reactions, despite the presence of several risk factors (Hammond et al., 2022; Line et al., 2022).

## 6 Clinical implications of pharmacogenomic testing

Pharmacogenomic (PGx) testing is playing an increasingly important role in psychiatric care, especially in preventing ADRs and optimizing treatment outcomes. One of its most promising applications is pre-emptive genetic screening to reduce the risk of DHRs and improve the tolerability of psychotropic medications.

### 6.1 Personalized prescribing through genetic screening

Pre-emptive PGx testing allows for the identification of genetic variants—particularly in drug-metabolizing enzymes such as CYP2D6 and CYP2C19—before initiating therapy. This information enables clinicians to tailor treatment strategies to an individual’s metabolic profile, reducing the likelihood of ADRs and improving therapeutic efficacy (Skokou et al., 2024).

Recent studies underscore the clinical benefits of PGx-guided psychiatric treatment. Compared to standard prescribing approaches, PGx-informed therapy has been associated with reduced incidence of ADRs, particularly nervous system-related effects such as sedation, insomnia, restlessness, and extrapyramidal symptoms. A 33.3% ADR incidence rate in PGx-guided patients, compared to 44.3% in the control group (Skokou et al., 2024). Reduced polypharmacy and fewer psychiatric hospitalizations, highlighting both improved patient safety and healthcare resource efficiency. Supporting these findings, studies reported a reduction in psychotropic medication use—including benzodiazepines and SSRIs—among patients treated according to PGx profiles, indicating greater drug stability and tolerability (Roberts et al., 2023; Scherf-Clavel et al., 2023; Westergaard et al., 2020).

Real-world cases further demonstrate the value of pharmacogenomic screening: A 75-year-old patient developed severe hepatotoxicity after starting Agomelatine. Despite normal liver function at baseline, PGx testing revealed the *CYP1A2* rs762551 AA genotype—linked to ultra-rapid metabolism and the accumulation of toxic metabolites (Wang et al., 2021). There are no CPIC guidelines for Agomelatine. Only a small PK study showed *CYP1A2* polymorphisms affect agomelatine clearance; in addition to case reports that demonstrated a link between *CYP1A2* variants and the potential drug-induced liver injury (DILI), level C (Song et al., 2014; Wang et al., 2021).

The metabolism of newer agents, such as cariprazine, is also influenced by pharmacogenomic factors. Cariprazine is primarily metabolized by CYP3A4, with minimal CYP2D6 involvement. A clinical study involving co-administration with erythromycin (a moderate CYP3A4 inhibitor) demonstrated increased cariprazine plasma concentrations, necessitating potential dose adjustments. Importantly, *CYP2D6* genotyping had no significant influence on its pharmacokinetics, suggesting it may not be necessary in this context (Tsermpini et al., 2022). Currently, there is no published pharmacogenomic evidence directly linking CYP variants to ADRs of cariprazine. However, the drug is metabolised by CYP3A4, and, to a lesser extent, CYP2D6, which makes drug–drug interactions clinically relevant (generally, level B for drugs metabolized by *CYP3A4*).

### 6.2 Guidelines supporting PGx implementation

Two major international bodies have established widely adopted PGx guidelines:

a. The Clinical Pharmacogenetics Implementation Consortium (CPIC) provides peer-reviewed recommendations and categorizes gene-drug pairs by actionability: Level A: Genetic data should guide therapy. Level B: Genetic data may be used to guide therapy. Levels C and D: Genetic data not currently actionable (Caudle et al., 2017; Morris et al., 2022).
b. The Dutch Pharmacogenetics Working Group (DPWG) offers gene-drug guidance integrated into European e-prescribing platforms. It ranks evidence quality (0–4) and clinical relevance (AA–F) to inform dosage, drug selection, and monitoring (Swen et al., 2011).

### 6.3 Integration of PGx in the UAE healthcare system

In the United Arab Emirates (UAE), PGx is gaining traction. The Department of Health–Abu Dhabi has incorporated PGx reporting *via* the Malaffi health information exchange system to support individualized prescribing. Priority is given to patients over 40 with a history of ADRs or treatment failure (Department of Health–Abu Dhabi, 2024). A pilot study in the UAE demonstrated the cost-effectiveness and clinical benefit of PGx testing for cardiovascular drugs (Al-Mahayri et al., 2022), suggesting its potential wider applicability to certain medications. However, limitations persist, including a lack of national PGx protocol, limited clinician awareness and training, and gaps in population-specific allele frequency data.

## 7 Challenges and limitations

Despite the growing promise of pharmacogenomics in improving drug safety and therapeutic outcomes, several significant challenges and limitations continue to hinder its widespread clinical adoption. One major obstacle is the variability in the availability and interpretation of genetic testing (Bousman et al., 2021). Access to pharmacogenomic testing remains uneven across different healthcare systems and regions, often limited to well-resourced or urban settings. Even when tests are available, inconsistencies in test platforms, reporting formats, and the interpretation of results can lead to confusion among healthcare providers. This variability reduces the reliability and clinical utility of test outcomes and contributes to hesitation in using pharmacogenomic data for therapeutic decisions.

Another critical concern involves the ethical and economic implications of pharmacogenomic testing (Karamperis et al., 2021; Pardiñas et al., 2021). From an ethical standpoint, questions about patient privacy, data security, and potential genetic discrimination remain unresolved. Genetic data is inherently sensitive, and its misuse could lead to social stigma or discrimination in insurance and employment. Economically, the high upfront cost of testing and the lack of reimbursement by many insurance systems pose substantial barriers. Although PGx testing may lead to long-term healthcare savings by reducing adverse drug reactions and improving treatment efficacy, the initial expense can deter healthcare institutions from incorporating these services, especially in lower-income or resource-constrained settings.

Furthermore, the lack of standardized guidelines and protocols for pharmacogenomic implementation continues to limit its integration into routine care (Alchakee et al., 2022; Kabbani et al., 2023). While organizations like CPIC and DPWG offer valuable recommendations, these are not uniformly adopted or enforced across national or institutional frameworks. Many healthcare systems lack cohesive policies regarding when and how to perform genetic testing, how to interpret the results, and how to apply them to prescribing decisions. This inconsistency contributes to underutilization and clinical uncertainty, as physicians may lack both the confidence and the tools to translate pharmacogenomic data into actionable insights.

Together, these challenges highlight the need for improving infrastructure, regulatory frameworks, clinician education, and cross-disciplinary collaboration to support the responsible, equitable, and effective integration of pharmacogenomics into clinical practice.

## 8 Conclusion and future directions

This review examined the underlying mechanisms of drug hypersensitivity reactions, with a particular focus on immunological pathways, the formation of reactive drug metabolites, and genetic susceptibility factors. These include well-established associations with HLA alleles and polymorphisms in drug-metabolizing enzymes such as those in the cytochrome P450 family. We explored the pharmacogenomics of key psychiatric medications, particularly anticonvulsants (e.g., carbamazepine, lamotrigine), antidepressants (e.g., SSRIs and novel agents), and antipsychotics (e.g., clozapine and newer generation drugs)—and identified several genetic variants linked to increased risk of adverse drug reactions. The clinical utility of pharmacogenomic testing was emphasized, especially in its capacity to pre-emptively identify individuals at high risk for DHRs and guide safer, more personalized prescribing practices. Nevertheless, several challenges persist, including the variability in test availability, differences in interpretation standards, ethical concerns surrounding genetic data use, and the absence of universally accepted guidelines for pharmacogenomic implementation.

Looking ahead, recent advances in genomic technology, particularly next-generation sequencing (NGS), have transformed the field by enabling the rapid and cost-effective analysis of genetic variation across diverse populations (Behjati and Tarpey, 2013). Beyond individual gene-drug interactions, NGS provides a foundational platform for multiomic approaches that integrate genomic, epigenomic, transcriptomic, proteomic, and metabolomic data to yield a comprehensive view of disease biology and drug response (Hasin et al., 2017). These multilayered datasets offer enhanced predictive power over single-layer genomic analyses, particularly for complex psychiatric disorders where treatment outcomes are influenced by diverse biological systems. A recent multiomic study in schizophrenia exemplifies this promise. By integrating DNA methylation data with genetic risk scores, researchers developed a robust predictive model for antipsychotic drug (APD) response, using epigenetic and genetic markers from six cortical genes. Validated in over 3,600 patients, this model demonstrated strong accuracy and clinical applicability, underscoring the potential of multiomic pharmacogenomics in personalizing psychiatric treatment (Guo et al., 2023).

Regionally, initiatives like the Emirati Genome Program are laying the groundwork for precision medicine by cataloging population-specific genetic variants through large-scale NGS efforts (Ateia et al., 2023). This localized reference data is crucial for tailoring pharmacogenomic applications to regional populations and may eventually support future multiomic analyses to refine clinical decision-making.

To maximize the global impact of these developments, international collaboration and equitable data sharing are essential. Pharmacogenomic insights must be drawn from diverse populations to ensure applicability and fairness in precision medicine (Relling and Klein, 2011). Open-access resources such as PharmGKB and collaborative efforts from CPIC and DPWG play pivotal roles in translating genomic evidence into clinical guidance. However, global uptake of these frameworks remains inconsistent. Expanding participation through international partnerships and prioritizing research in underrepresented populations will be key. By developing shared, globally accessible pharmacogenomic databases, we can transform localized discoveries into scalable, real-world tools for personalized psychiatric care.

In conclusion, pharmacogenomics holds transformative potential to optimize psychiatric drug therapy, reduce adverse reactions, and improve treatment outcomes. Continued investment in research, infrastructure, and international collaboration will be vital to translating this promise into routine clinical practice worldwide.

## References

[B1] Adachi A. Komine M. Tsuda H. Nakajima S. Kabashima K. Ohtsuki M. (2019). Differential expression of alarmins: IL-33 as a candidate marker for early diagnosis of toxic epidermal necrolysis. J. Allergy Clin. Immunol. Pract. 7 (1), 325–327. 10.1016/j.jaip.2018.05.037 29936189

[B2] Adair K. Meng X. Naisbitt D. J. (2021). Drug hapten‐specific t‐cell activation: current status and unanswered questions. PROTEOMICS 21 (17–18), e2000267. 10.1002/pmic.202000267 33651918

[B3] Ahmed A. F. Sukasem C. Sabbah M. A. Musa N. F. Mohamed Noor D. A. Daud N. A. A. (2021). Genetic determinants in HLA and cytochrome P450 genes in the risk of aromatic antiepileptic-induced severe cutaneous adverse reactions. J. Personalized Med. 11 (5), 383. 10.3390/jpm11050383 34067134 PMC8150699

[B5] Al-Mahayri Z. N. Khasawneh L. Q. Alqasrawi M. N. Altoum S. M. Jamil G. Badawi S. (2022). Pharmacogenomics implementation in cardiovascular disease in a highly diverse population: initial findings and lessons learned from a pilot study in United Arab Emirates. Hum. Genomics 16 (1), 42. 10.1186/s40246-022-00417-9 36154845 PMC9509637

[B6] Alchakee A. Ahmed M. Eldohaji L. Alhaj H. Saber-Ayad M. (2022). Pharmacogenomics in psychiatry practice: the value and the challenges. Int. J. Mol. Sci. 23 (21), 13485. 10.3390/ijms232113485 36362270 PMC9655367

[B7] Alfirevic A. Pirmohamed M. (2010). Drug induced hypersensitivity and the HLA complex. Pharmaceuticals 4 (1), 69–90. 10.3390/ph4010069

[B8] Alhaj H. A. McAllister-Williams R. H. (2008). “Chapter 5.7 adrenal steroids and episodic memory: relevance to mood disorders,” in Chapter 5.7 adrenal steroids and episodic memory: relevance to mood disorders, 18, 585–595. 10.1016/S1569-7339(08)00232-4

[B9] Alhaj H. A. Massey A. E. McAllister-Williams R. H. (2008). Effects of cortisol on the laterality of the neural correlates of episodic memory. J. Psychiatric Res. 42 (12), 971–981. 10.1016/j.jpsychires.2007.11.008 18187154

[B10] Alhaj H. A. Selman M. Jervis V. Rodgers J. Barton S. McAllister-Williams R. H. (2012). Effect of low-dose acute tryptophan depletion on the specificity of autobiographical memory in healthy subjects with a family history of depression. Psychopharmacology 222 (2), 285–292. 10.1007/s00213-012-2644-x 22286957

[B11] Almeida C.-A. van Miert P. O’Driscoll K. Zoet Y. M. Chopra A. Witt C. (2019). Virus-specific T-cell clonotypes might contribute to drug hypersensitivity reactions through heterologous immunity. J. Allergy Clin. Immunol. 144 (2), 608–611.e4. 10.1016/j.jaci.2019.05.009 31102700

[B12] Anaya J.-M. Shoenfeld Y. Rojas-Villarraga A. Levy R. A. Cervera R. (2013). Autoimmunity: from bench to bedside. 29087650

[B13] Andrews M. H. Matthews S. G. (2004). Programming of the hypothalamo–pituitary–adrenal axis: serotonergic involvement. Stress 7 (1), 15–27. 10.1080/10253890310001650277 15204029

[B14] Ateia H. Ogrodzki P. Wilson H. V. Ganesan S. Halwani R. Koshy A. (2023). Population genome programs across the Middle East and North Africa: successes, challenges, and future directions. Biomed. Hub. 8 (1), 60–71. 10.1159/000530619 37900972 PMC10601860

[B15] Athanasiu L. Smorr L.-L. H. Tesli M. Røssberg J. I. Sønderby I. E. Spigset O. (2015). Genome-wide association study identifies common variants associated with pharmacokinetics of psychotropic drugs. J. Psychopharmacol. 29 (8), 884–891. 10.1177/0269881115584469 25944848

[B16] Azoury M. E. Filì L. Bechara R. Scornet N. de Chaisemartin L. Weaver R. J. (2018). Identification of T‐cell epitopes from benzylpenicillin conjugated to human serum albumin and implication in penicillin allergy. Allergy 73 (8), 1662–1672. 10.1111/all.13418 29355985

[B17] Bajwa S. F. Mohammed R. H. (2025). Type II hypersensitivity reaction. 33085411

[B18] Behjati S. Tarpey P. S. (2013). What is next generation sequencing? Archives Dis. Child. - Educ. and Pract. Ed. 98 (6), 236–238. 10.1136/archdischild-2013-304340 23986538 PMC3841808

[B19] Böhm R. Cascorbi I. (2016). Pharmacogenetics and predictive testing of drug hypersensitivity reactions. Front. Pharmacol. 7, 396. 10.3389/fphar.2016.00396 27818635 PMC5073094

[B20] Bousman C. A. Bengesser S. A. Aitchison K. J. Amare A. T. Aschauer H. Baune B. T. (2021). Review and consensus on pharmacogenomic testing in psychiatry. Pharmacopsychiatry 54 (01), 5–17. 10.1055/a-1288-1061 33147643 PMC13579664

[B21] Cacabelos R. Cacabelos P. Aliev G. (2013). Genomics of schizophrenia and pharmacogenomics of antipsychotic drugs. Open J. Psychiatry 03 (01), 46–139. 10.4236/ojpsych.2013.31008

[B22] Calabrese F. Molteni R. Racagni G. Riva M. A. (2009). Neuronal plasticity: a link between stress and mood disorders. Psychoneuroendocrinology 34, S208–S216. 10.1016/j.psyneuen.2009.05.014 19541429

[B23] Cardona V. Guilarte M. Luengo O. Labrador-Horrillo M. Sala-Cunill A. Garriga T. (2011). Allergic diseases in the elderly. Clin. Transl. Allergy 1 (1), 11. 10.1186/2045-7022-1-11 22409889 PMC3339328

[B24] Carr D. F. Pirmohamed M. (2018). Biomarkers of adverse drug reactions. Exp. Biol. Med. 243 (3), 291–299. 10.1177/1535370217733425 28950720 PMC5813863

[B25] Castrejon J. L. Berry N. El-Ghaiesh S. Gerber B. Pichler W. J. Park B. K. (2010). Stimulation of human T cells with sulfonamides and sulfonamide metabolites. J. Allergy Clin. Immunol. 125 (2), 411–418. 10.1016/j.jaci.2009.10.031 20159253

[B99] Caudle K. E. Dunnenberger H. M. Freimuth R. R. Peterson J. F. Burlison J. D. Whirl-Carrillo M. (2017). Standardizing terms for clinical pharmacogenetic test results: consensus terms from the clinical pharmacogenetics implementation consortium (CPIC). Genet. Med. 19 (2), 215–223. 10.1038/gim.2016.87 27441996 PMC5253119

[B26] Chowdhury D. Chin L. Odabashian R. Fawaz A. Canil C. Ong M. (2025). Diagnosis and management of skin toxicities in systemic treatment of genitourinary cancers. Cancers 17 (2), 251. 10.3390/cancers17020251 39858032 PMC11763385

[B27] Clayden R. C. Zaruk A. Meyre D. Thabane L. Samaan Z. (2012). The association of attempted suicide with genetic variants in the SLC6A4 and TPH genes depends on the definition of suicidal behavior: a systematic review and meta-analysis. Transl. Psychiatry 2 (10), e166. 10.1038/tp.2012.96 23032942 PMC3565826

[B100] Crux N. B. Elahi S. (2017). Human leukocyte antigen (HLA) and immune regulation: how do classical and non-classical HLA Alleles Modulate Immune Response to Human Immunodeficiency Virus and Hepatitis C Virus Infections?. Front Immunol. 18 (8), 832. 10.3389/fimmu.2017.00832 28769934 PMC5513977

[B28] De la Cruz-Cano E. (2017). Association between FKBP5 and CRHR1 genes with suicidal behavior: a systematic review. Behav. Brain Res. 317, 46–61. 10.1016/j.bbr.2016.09.032 27638035

[B101] Del Pozzo-Magaña B. R. Liy-Wong C. (2024). A concise review of cutaneous adverse drug reactions. Br. J. Clin. Pharmacol. 90 (8), 1838–1855. 10.1111/bcp.15490 35974692

[B29] Elmokadem A. Bruno C. D. Housand C. Jordie E. B. Chow C. R. Lesko L. J. (2022). Brexpiprazole pharmacokinetics in CYP2D6 poor metabolizers: using physiologically based pharmacokinetic modeling to optimize time to effective concentrations. J. Clin. Pharmacol. 62 (1), 66–75. 10.1002/jcph.1946 34328221

[B30] Elzagallaai A. A. Rieder M. J. (2022). Genetic markers of drug hypersensitivity in pediatrics: current state and promise. Expert Rev. Clin. Pharmacol. 15 (6), 715–728. 10.1080/17512433.2022.2100345 35811487

[B31] Frederiksen T. Areberg J. Raoufinia A. Schmidt E. Stage T. B. Brøsen K. (2023). Estimating the *in vivo* function of *CYP2D6* alleles through population pharmacokinetic modeling of brexpiprazole. Clin. Pharmacol. and Ther. 113 (2), 360–369. 10.1002/cpt.2791 36350097 PMC10099095

[B32] Fujita H. Matsukura S. Watanabe T. Komitsu N. Watanabe Y. Takahashi Y. (2014). The serum level of HMGB1 (high mobility group box 1 protein) is preferentially high in drug-induced hypersensitivity syndrome/drug reaction with eosinophilia and systemic symptoms. Br. J. Dermatology 171 (6), 1585–1588. 10.1111/bjd.13162 24903194

[B33] Goldstein J. I. Jarskog L. F. Hilliard C. Alfirevic A. Duncan L. Fourches D. (2014). Clozapine-induced agranulocytosis is associated with rare HLA-DQB1 and HLA-B alleles. Nat. Commun. 5, 4757. 10.1038/ncomms5757 25187353 PMC4155508

[B34] González-Galarza F. F. Takeshita L. Y. C. Santos E. J. M. Kempson F. Maia M. H. T. Silva A. L. S. da (2015). Allele frequency net 2015 update: new features for HLA epitopes, KIR and disease and HLA adverse drug reaction associations. Nucleic Acids Res. 43 (D1), D784–D788. 10.1093/nar/gku1166 25414323 PMC4383964

[B35] Graudins L. V. Trubiano J. A. Zubrinich C. M. Elliott A. S. Aung A. K. (2018). Medication‐related anaphylaxis treated in hospital: agents implicated, patient outcomes, and management lessons. Pharmacoepidemiol. Drug Saf. 27 (9), 1029–1033. 10.1002/pds.4587 30051944

[B36] Guo L.-K. Su Y. Zhang Y.-Y.-N. Yu H. Lu Z. Li W.-Q. (2023). Prediction of treatment response to antipsychotic drugs for precision medicine approach to schizophrenia: randomized trials and multiomics analysis. Mil. Med. Res. 10 (1), 24. 10.1186/s40779-023-00459-7 37269009 PMC10236828

[B37] Hammond S. Thomson P. Meng X. Naisbitt D. (2021). *In-Vitro* approaches to predict and study T-Cell mediated hypersensitivity to drugs. Front. Immunol. 12, 630530. 10.3389/fimmu.2021.630530 33927714 PMC8076677

[B38] Hammond S. Olsson-Brown A. Grice S. Gibson A. Gardner J. Castrejón-Flores J. L. (2022). Checkpoint inhibition reduces the threshold for drug-specific T-Cell priming and increases the incidence of sulfasalazine hypersensitivity. Toxicol. Sci. 186 (1), 58–69. 10.1093/toxsci/kfab144 34850240 PMC8883351

[B39] Harr T. French L. E. (2010). Toxic epidermal necrolysis and Stevens-Johnson syndrome. Orphanet J. Rare Dis. 5 (1), 39. 10.1186/1750-1172-5-39 21162721 PMC3018455

[B40] Hasin Y. Seldin M. Lusis A. (2017). Multi-omics approaches to disease. Genome Biol. 18 (1), 83. 10.1186/s13059-017-1215-1 28476144 PMC5418815

[B41] Hung S.-I. Mockenhaupt M. Blumenthal K. G. Abe R. Ueta M. Ingen-Housz-Oro S. (2024). Severe cutaneous adverse reactions. Nat. Rev. Dis. Prim. 10 (1), 30. 10.1038/s41572-024-00514-0 38664435 PMC13052379

[B42] Islam F. Hain D. Lewis D. Law R. Brown L. C. Tanner J.-A. (2022). Pharmacogenomics of clozapine-induced agranulocytosis: a systematic review and meta-analysis. Pharmacogenomics J. 22 (4), 230–240. 10.1038/s41397-022-00281-9 35710824 PMC9363274

[B43] Kabbani D. Akika R. Wahid A. Daly A. K. Cascorbi I. Zgheib N. K. (2023). Pharmacogenomics in practice: a review and implementation guide. Front. Pharmacol. 14, 1189976. 10.3389/fphar.2023.1189976 37274118 PMC10233068

[B44] Kalesnikoff J. Galli S. J. (2008). New developments in mast cell biology. Nat. Immunol. 9 (11), 1215–1223. 10.1038/ni.f.216 18936782 PMC2856637

[B45] Kaniwa N. Saito Y. (2013). The risk of cutaneous adverse reactions among patients with the *HLA-A* 31:01* allele who are given carbamazepine, oxcarbazepine or eslicarbazepine: a perspective review. Ther. Adv. Drug Saf. 4 (6), 246–253. 10.1177/2042098613499791 25114785 PMC4125310

[B46] Karamperis K. Koromina M. Papantoniou P. Skokou M. Kanellakis F. Mitropoulos K. (2021). Economic evaluation in psychiatric pharmacogenomics: a systematic review. Pharmacogenomics J. 21 (4), 533–541. 10.1038/s41397-021-00249-1 34215853

[B47] Kim S.-H. Naisbitt D. J. (2016). Update on advances in research on idiosyncratic drug-induced liver injury. Allergy, Asthma and Immunol. Res. 8 (1), 3–11. 10.4168/aair.2016.8.1.3 26540496 PMC4695405

[B48] Kim S.-H. Lee K. W. Song W.-J. Kim S.-H. Jee Y.-K. Lee S.-M. (2011). Carbamazepine-induced severe cutaneous adverse reactions and HLA genotypes in koreans. Epilepsy Res. 97 (1–2), 190–197. 10.1016/j.eplepsyres.2011.08.010 21917426

[B49] Koomdee N. Pratoomwun J. Jantararoungtong T. Theeramoke V. Tassaneeyakul W. Klaewsongkram J. (2017). Association of HLA-A and HLA-B alleles with Lamotrigine-Induced cutaneous adverse drug reactions in the Thai population. Front. Pharmacol. 8, 879. 10.3389/fphar.2017.00879 29238301 PMC5712579

[B50] Land W. G. (2023). “Perspectives of the danger/injury model of immunology as applied to antigen-related human disorders,” in Damage-associated molecular patterns in human diseases (Springer International Publishing), 3–44. 10.1007/978-3-031-21776-0_1

[B51] Langmia I. M. Just K. S. Yamoune S. Müller J. P. Stingl J. C. (2022). Pharmacogenetic and drug interaction aspects on ketamine safety in its use as antidepressant - implications for precision dosing in a global perspective. Br. J. Clin. Pharmacol. 88 (12), 5149–5165. 10.1111/bcp.15467 35863300

[B52] Li Y. Deshpande P. Hertzman R. J. Palubinsky A. M. Gibson A. Phillips E. J. (2021). Genomic risk factors driving immune-mediated delayed drug hypersensitivity reactions. Front. Genet. 12, 641905. 10.3389/fgene.2021.641905 33936169 PMC8085493

[B53] Line J. Thomson P. Naisbitt D. J. (2022). Pathology of T-cell-mediated drug hypersensitivity reactions and impact of tolerance mechanisms on patient susceptibility. Curr. Opin. Allergy and Clin. Immunol. 22 (4), 226–233. 10.1097/ACI.0000000000000834 35779063

[B54] Locharernkul C. Loplumlert J. Limotai C. Korkij W. Desudchit T. Tongkobpetch S. (2008). Carbamazepine and phenytoin induced stevens‐johnson syndrome is associated with HLA‐B*1502 allele in Thai population. Epilepsia 49 (12), 2087–2091. 10.1111/j.1528-1167.2008.01719.x 18637831

[B55] Mahmood T. El-Asrag M. E. Poulter J. A. Cardno A. G. Tomlinson A. Ahmed S. (2021). A recessively inherited risk locus on chromosome 13q22-31 conferring susceptibility to schizophrenia. Schizophr. Bull. 47 (3), 796–802. 10.1093/schbul/sbaa161 33159203 PMC8084434

[B56] Malekpour M. Shekouh D. Safavinia M. E. Shiralipour S. Jalouli M. Mortezanejad S. (2023). Role of FKBP5 and its genetic mutations in stress-induced psychiatric disorders: an opportunity for drug discovery. Front. Psychiatry 14, 1182345. 10.3389/fpsyt.2023.1182345 37398599 PMC10313426

[B57] Masmoudi H. C. Afify N. Alnaqbi H. Alhalwachi Z. Tay G. K. Alsafar H. (2022). HLA pharmacogenetic markers of drug hypersensitivity from the perspective of the populations of the greater Middle East. Pharmacogenomics 23 (12), 695–708. 10.2217/pgs-2022-0078 35971864

[B58] McAllister-Williams R. H. Alhaj H. A. Massey A. Pankiv J. Reckermann U. (2014). Somatodendritic 5-hydroxytryptamine_1A_ (5-HT_1A_) autoreceptor function in major depression as assessed using the shift in electroencephalographic frequency spectrum with buspirone. Psychol. Med. 44 (4), 767–777. 10.1017/S0033291713001475 23809646

[B59] McCormack M. Alfirevic A. Bourgeois S. Farrell J. J. Kasperavičiūtė D. Carrington M. (2011). HLA-A*3101 and carbamazepine-induced hypersensitivity reactions in Europeans. N. Engl. J. Med. 364 (12), 1134–1143. 10.1056/NEJMoa1013297 21428769 PMC3113609

[B60] Meng X. Yerly D. Naisbitt D. J. (2018). Mechanisms leading to T-cell activation in drug hypersensitivity. Curr. Opin. Allergy and Clin. Immunol. 18 (4), 317–324. 10.1097/ACI.0000000000000458 29905574

[B61] Mockenhaupt M. (2017). Epidemiology of cutaneous adverse drug reactions. Allergol. Sel. 1 (1), 96–108. 10.5414/ALX01508E 30402608 PMC6039997

[B103] Morris S. A. Alsaidi A. T. Verbyla A. Cruz A. Macfarlane C. Bauer J. (2022). Cost effectiveness of pharmacogenetic testing for drugs with clinical pharmacogenetics implementation consortium (CPIC) guidelines: a systematic review. Clin. Pharmacol. Ther. 112 (6), 1318–1328. 10.1002/cpt.2754 36149409 PMC9828439

[B102] Moore M. J. Sathe N. C. Ganipisetti V. M. (2023). Acute Generalized Exanthematous Pustulosis. Acute generalized exanthematous pustulosis. Treasure Island, FL: StatPearls Publishing. Available online at: https://www.ncbi.nlm.nih.gov/books/NBK592407/?utm_source (Accessed September 9, 2025) 37276304

[B63] Muzumdar S. Rothe M. J. Grant-Kels J. M. (2019). The rash with maculopapules and fever in children. Clin. Dermatology 37 (2), 119–128. 10.1016/j.clindermatol.2018.12.005 30981292

[B64] Naisbitt D. J. Gordon S. Pirmohamed M. Park B. (2000). Immunological principles of adverse drug reactions: the initiation and propagation of immune responses elicited by drug treatment. Drug Saf. 23 (6), 483–507. 10.2165/00002018-200023060-00002 11144658

[B65] Naisbitt D. J. Nattrass R. G. Ogese M. O. (2014). *In vitro* diagnosis of delayed-type drug hypersensitivity: mechanistic aspects and unmet needs. Immunol. Allergy Clin. N. Am. 34 (3), 691–705. 10.1016/j.iac.2014.04.009 25017686

[B66] Nakajima S. Watanabe H. Tohyama M. Sugita K. Iijima M. Hashimoto K. (2011). High-mobility group box 1 protein (HMGB1) as a novel diagnostic tool for toxic epidermal necrolysis and Stevens-Johnson syndrome. Archives Dermatology 147 (9), 1110–1112. 10.1001/archdermatol.2011.239 21931056

[B68] Ostrov D. A. Grant B. J. Pompeu Y. A. Sidney J. Harndahl M. Southwood S. (2012). Drug hypersensitivity caused by alteration of the MHC-Presented self-peptide repertoire. Proc. Natl. Acad. Sci. 109 (25), 9959–9964. 10.1073/pnas.1207934109 22645359 PMC3382472

[B69] Pallardy M. Bechara R. (2017). Chemical or drug hypersensitivity: is the immune system clearing the danger? Toxicol. Sci. 158 (1), 14–22. 10.1093/toxsci/kfx084 28472426

[B70] Pardiñas A. F. Owen M. J. Walters J. T. R. (2021). Pharmacogenomics: a road ahead for precision medicine in psychiatry. Neuron 109 (24), 3914–3929. 10.1016/j.neuron.2021.09.011 34619094

[B71] Pattanaik S. Jain A. Ahluwalia J. (2021). Evolving role of pharmacogenetic biomarkers to predict drug-induced hematological disorders. Ther. Drug Monit. 43 (2), 201–220. 10.1097/FTD.0000000000000842 33235023

[B72] Pavlos R. Mallal S. Phillips E. (2012). HLA and pharmacogenetics of drug hypersensitivity. Pharmacogenomics 13 (11), 1285–1306. 10.2217/pgs.12.108 22920398

[B73] Periclou A. Phillips L. Ghahramani P. Kapás M. Carrothers T. Khariton T. (2021). Population pharmacokinetics of cariprazine and its major metabolites. Eur. J. Drug Metabolism Pharmacokinet. 46 (1), 53–69. 10.1007/s13318-020-00650-4 33141308 PMC7811992

[B74] Pichler W. J. (2019). Immune pathomechanism and classification of drug hypersensitivity. Allergy 74 (8), 1457–1471. 10.1111/all.13765 30843233

[B75] Pirmohamed M. Naisbitt D. J. Gordon F. Park B. K. (2002). The danger Hypothesis—potential role in idiosyncratic drug reactions. Toxicology 181 (182), 55–63. 10.1016/S0300-483X(02)00255-X 12505285

[B76] Posadas S. J. Pichler W. J. (2007). Delayed drug hypersensitivity reactions – new concepts. Clin. and Exp. Allergy 37 (7), 989–999. 10.1111/j.1365-2222.2007.02742.x 17581192

[B77] Relling M. V. Klein T. E. (2011). CPIC: clinical pharmacogenetics implementation consortium of the pharmacogenomics research network. Clin. Pharmacol. and Ther. 89 (3), 464–467. 10.1038/clpt.2010.279 21270786 PMC3098762

[B78] Roberts B. Cooper Z. Lu S. Stanley S. Majda B. T. Collins K. R. L. (2023). Utility of pharmacogenetic testing to optimise antidepressant pharmacotherapy in youth: a narrative literature review. Front. Pharmacol. 14, 1267294. 10.3389/fphar.2023.1267294 37795032 PMC10545970

[B79] Roujeau J.-C. (2005). Clinical heterogeneity of drug hypersensitivity. Toxicology 209 (2), 123–129. 10.1016/j.tox.2004.12.022 15767024

[B80] Scherf-Clavel M. Frantz A. Eckert A. Weber H. Unterecker S. Deckert J. (2023). Effect of CYP2D6 pharmacogenetic phenotype and phenoconversion on serum concentrations of antidepressants and antipsychotics: a retrospective cohort study. Int. J. Clin. Pharm. 45 (5), 1107–1117. 10.1007/s11096-023-01588-8 37166747 PMC10600053

[B81] Šimić I. Potočnjak I. Kraljičković I. Stanić Benić M. Čegec I. Juričić Nahal D. (2016). *CYP2D6 *6/*6* genotype and drug interactions as cause of haloperidol-induced extrapyramidal symptoms. Pharmacogenomics 17 (13), 1385–1389. 10.2217/pgs-2016-0069 27469576

[B82] Skokou M. Karamperis K. Koufaki M.-I. Tsermpini E.-E. Pandi M.-T. Siamoglou S. (2024). Clinical implementation of preemptive pharmacogenomics in psychiatry. EBioMedicine 101, 105009. 10.1016/j.ebiom.2024.105009 38364700 PMC10879811

[B83] Song L. Du Q. Jiang X. Wang L. (2014). Effect of *CYP1A2* polymorphism on the pharmacokinetics of agomelatine in Chinese healthy male volunteers. J. Clin. Pharm. Ther. 39 (2), 204–209. 10.1111/jcpt.12118 24372004

[B104] Swen J. J. Nijenhuis M. De Boer A. Grandia L. Maitland-Van Der Zee A. H. Mulder H. (2011). Pharmacogenetics: From bench to byte an update of guidelines. Clin. Pharmacol. Ther. 89 (5), 662–673. 10.1038/clpt.2011.34 21412232

[B84] Szabó M. Hujber Z. Harsányi J. Szatmári B. Dombi Z. B. Magyar G. (2024). Coadministration of cariprazine with a moderate CYP3A4 inhibitor in patients with schizophrenia: implications for dose adjustment and safety monitoring. Clin. Pharmacokinet. 63 (10), 1501–1510. 10.1007/s40262-024-01431-x 39427282 PMC11522146

[B85] Szegedi A. Remenyik É. Gellén E. (2023). “Drug hypersensitivity reactions,” in European handbook of dermatological treatments (Springer International Publishing), 229–245. 10.1007/978-3-031-15130-9_22

[B86] Tailor A. Waddington J. C. Meng X. Park B. K. (2016). Mass spectrometric and functional aspects of drug–protein conjugation. Chem. Res. Toxicol. 29 (12), 1912–1935. 10.1021/acs.chemrestox.6b00147 27689879

[B87] Tsermpini E. E. Terzi T. Kores Plesničar B. Dolžan V. (2022). “Pharmacogenomics and antipsychotics: efficacy and adverse drug reactions,” in Psychiatric genomics (Elsevier), 161–188. 10.1016/B978-0-12-819602-1.00010-3

[B88] Uzzaman A. Cho S. H. (2012). Chapter 28: classification of hypersensitivity reactions. Allergy Asthma Proc. 33 (3), 96–99. 10.2500/aap.2012.33.3561 22794701

[B89] Verheyden M. Murrell D. Bilgic A. (2020). 12871 A systematic review of drug-induced pemphigoid. J. Am. Acad. Dermatology 83 (6), AB113. 10.1016/j.jaad.2020.06.539 PMC9207627 32176310

[B90] Vickers M. Ramineni V. Malacova E. Eriksson L. McMahon K. Moudgil V. (2022). Risk factors for clozapine-induced myocarditis and cardiomyopathy: a systematic review and meta-analysis. Acta Psychiatr. Scand. 145 (5), 442–455. 10.1111/acps.13398 35067911

[B91] Wang Q. Zhou J. Zhou L. Chen Z. Fang Z. Chen S. (2011). Association between HLA-B*1502 allele and carbamazepine-induced severe cutaneous adverse reactions in Han people of southern China mainland. Seizure 20 (6), 446–448. 10.1016/j.seizure.2011.02.003 21397523

[B92] Wang S. Xu Q. Qu K. Wang J. Zhou Z. (2021). CYP1A2 polymorphism may contribute to agomelatine-induced acute liver injury: case report and review of the literature. Medicine 100 (45), e27736. 10.1097/MD.0000000000027736 34766583 PMC10545369

[B93] Wei B. M. Fox L. P. Kaffenberger B. H. Korman A. M. Micheletti R. G. Mostaghimi A. (2024). Drug-induced hypersensitivity syndrome/drug reaction with eosinophilia and systemic symptoms. Part I. Epidemiology, pathogenesis, clinicopathological features, and prognosis. J. Am. Acad. Dermatology 90 (5), 885–908. 10.1016/j.jaad.2023.02.072 37516359

[B94] Westergaard N. Søgaard Nielsen R. Jørgensen S. Vermehren C. (2020). Drug use in Denmark for drugs having Pharmacogenomics (PGx) based Dosing Guidelines from CPIC or DPWG for CYP2D6 and CYP2C19 drug–gene pairs: perspectives for introducing PGx test to polypharmacy patients. J. Personalized Med. 10 (1), 3. 10.3390/jpm10010003 31963319 PMC7151550

[B95] White K. D. Chung W.-H. Hung S.-I. Mallal S. Phillips E. J. (2015). Evolving models of the immunopathogenesis of T cell–mediated drug allergy: the role of host, pathogens, and drug response. J. Allergy Clin. Immunol. 136 (2), 219–235. 10.1016/j.jaci.2015.05.050 26254049 PMC4577472

[B96] Wilkinson I. D. Mahmood T. Yasmin S. F. Tomlinson A. Nazari J. Alhaj H. (2023). In memory of professor iain wilkinson: cognitive and neuroimaging endophenotypes in a consanguineous schizophrenia multiplex family. Psychol. Med. 53 (7), 3178–3186. 10.1017/S0033291721005250 35125130 PMC10235651

[B97] Willemin M.-E. Zannikos P. Mannens G. de Zwart L. Snoeys J. (2022). Prediction of drug-drug interactions after esketamine intranasal administration using a physiologically based pharmacokinetic model. Clin. Pharmacokinet. 61 (8), 1115–1128. 10.1007/s40262-022-01123-4 35579824

[B98] Zhang X. Xiang Q. Zhao X. Ma L. Cui Y. (2019). Association between aripiprazole pharmacokinetics and CYP2D6 phenotypes: a systematic review and meta-analysis. J. Clin. Pharm. Ther. 44 (2), 163–173. 10.1111/jcpt.12780 30565279

